# Transcriptome analysis reveals the molecular mechanisms of adaptation to high temperatures in *Gracilaria bailinae*

**DOI:** 10.3389/fpls.2023.1125324

**Published:** 2023-04-14

**Authors:** Yongjian Huang, Jianjun Cui, Sipan Wang, Xinyi Chen, Jiawei Liao, Youyou Guo, Rong Xin, Bowen Huang, Enyi Xie

**Affiliations:** Fishery College, Guangdong Ocean University, Zhanjiang, China

**Keywords:** heat-tolerance mechanism, gene regulation, *Gracilaria bailinae*, macroalgae, temperature, transcriptome

## Abstract

Global warming causes great thermal stress to macroalgae and those species that can adapt to it are thought to be better able to cope with warmer oceans. *Gracilaria bailinae*, a macroalgae with high economic and ecological values, can survive through the hot summer in the South China Sea, but the molecular mechanisms underlying its adaptation to high temperatures are unclear. To address this issue, the present study analyzed the growth and transcriptome of *G. bailinae* after a 7-day exposure to 15°C (LT: low temperature), 25°C (MT: middle temperature), and 35°C (HT: high temperature). Growth analysis showed that the HT group had the highest relative growth rate (RGR = 2.1%) with the maximum photochemical quantum yield of PSII (*F*_v_/*F*_m_ = 0.62) remaining within the normal range. Transcriptome analysis showed more differentially expressed genes (DEGs) in the comparison between MT and HT groups than in that between MT and LT, and most of these DEGs tended to be downregulated at higher temperatures. The KEGG pathway enrichment analysis showed that the DEGs were mainly enriched in the carbohydrate, energy, and lipid metabolisms. In addition, the genes involved in NADPH and ATP synthesis, which are associated with photosynthesis, the Calvin cycle, pyruvate metabolism, and the citrate cycle, were downregulated. Downregulation was also observed in genes that encode enzymes involved in fatty acid desaturation and alpha-linolenic acid metabolism. In summary, *G. bailinae* regulated the synthesis of NADPH and ATP, which are involved in the above-mentioned processes, to reduce unnecessary energy consumption, and limited the synthesis of enzymes in the metabolism of unsaturated fatty acids and alpha-linolenic acid to adapt to high environmental temperatures. The results of this study improve our understanding of the molecular mechanisms underlying the adaptation of *G. bailinae* to high temperatures.

## 1 Introduction

Since the industrial revolution, increasing anthropogenic CO_2_ and other greenhouse gas emissions have resulted in an energy imbalance on Earth; specifically, in a positive radiative imbalance at the highest atmospheric layers (Hansen et al., 2011; Von Schuckmann et al., 2016; Association, 2022). As a result, energy began to accumulate as heat in the Earth system, which is what ultimately drives global warming (Hansen et al., 2011; Von Schuckmann et al., 2016; Association, 2022). The global average temperature in 2021 was approximately 1.11 ± 0.13°C warmer than the pre-industrial average recorded between 1850 and 1900, and the 2015–2021 period has been the hottest on record (Association, 2022). Our oceans have absorbed over 90% of the energy accumulated in the Earth system through human-induced greenhouse gas emissions, causing a rise in ocean heat content, which in 2021 was the highest on record (Association, 2022). The Special Report published by the Intergovernmental Panel on Climate Change in 2019 also noted that the oceans have warmed unabated since 2005 (Bindoff et al., 2019). In addition, the frequency of marine heatwaves (MHWs) has increased during the past century, with potential consequences for marine organisms and the communities that depend on them (Smale et al., 2019). Between 1982 and 2020, the duration and frequency of MHWs in the South China Sea increased significantly (Yao and Wang, 2021). The continued warming of the oceans is having an enormous impact on macroalgae. One of the most crucial aspects affecting their dispersion is temperature, since it has an influence on their physiology, growth, and adaptation (Graba-Landry et al., 2018; Ji and Gao, 2021). Ocean warming hinders the growth of some tropical brown algae and subtropical macroalgae, forces tropical species to exist at their maximum temperature limit, and can even cause the entire macroalgal community to retreat (Wernberg et al., 2011; Koch et al., 2013; Bender et al., 2014; Graba-Landry et al., 2018; Ji and Gao, 2021). Increasing sea surface temperatures have already led to the loss of important habitat-forming kelp and macroalgae (Graba-Landry et al., 2018). If global warming persists, the distribution of certain seaweeds will change even more, and some might go extinct (Wernberg et al., 2011; Yao and Somero, 2014). It is thought that macroalgal species that can adapt to heat stress would do better in the increasingly warmer oceans (Ji and Gao, 2021).

The ability of plants to sense temperature stimuli, generate and transmit signals, and activate the proper morpho-anatomical, physiological, and molecular responses plays a vital role in their survival in hot environments (Hasanuzzaman et al., 2013; Zhang et al., 2020). Algae generally employ a variety of coping and repair mechanisms, including the regulation of photosynthesis to balance energy output and consumption, the modification of membrane fluidity by altering the degree of fatty acid saturation, the accumulation of compatible solutes to keep cell osmolality constant, and the activation of the enzymatic antioxidant system to remove excessive reactive oxygen species (Barati et al., 2019). RNA sequencing (RNA-seq) is a comprehensive technique for analyzing gene function and revealing the molecular mechanisms behind certain biological processes (Lim et al., 2016). This technique has steadily been employed in the study of macroalgae’s molecular biology in recent years (Wang et al., 2015; Wenlei et al., 2018; Qin et al., 2021). For example, RNA-seq has been crucial in 1) tracking the expression of essential genes and critical metabolic processes in the growth and development of *Gracilariopsis lemaneiformis* (Wang et al., 2015; Qin et al., 2021), 2) investigating the molecular processes of *G. lemaneiformis’* reaction to low temperature environments (Qin et al., 2021), and 3) understanding *Pyropia haitanensis’* thermal adaptation to high temperatures (Wenlei et al., 2018).

*Gracilaria bailinae* is a red alga generally used for agar extraction, animal farming, and aquaculture water treatments (Elle et al., 2017; Xu et al., 2021). Specifically, this species has the potential to limit the spread of pathogenic *Vibrio*, reduce unionized ammonia, and promote the steady growth of advantageous phytoplankton (*Nannochloropsis* and *Chlorella*) (Elle et al., 2017). Additionally, due to its high agar yield of 29.8% dw (Skriptsova and Nabivailo, 2009), *G. bailinae* is greatly sought after for uses in the processed food, cosmetic, and pharmaceutical industries, and also in the fields of microbiology and molecular biology (Endoma et al., 2020). Preliminary studies found that *G. bailinae* can grow at a temperature range between 15 and 35°C and shows several traits associated with high-temperature tolerance (unpublished). Within the context of global warming, this species has a great potential for future applications as it can be farmed on a large scale all year round in tropical areas, continuously providing the raw materials for ecological restoration programs, sewage purification, agar and feed manufacturing, etc. However, the molecular mechanisms behind *G. bailinae’s* reaction to high temperatures remain unknown. The present study specifically aims to reveal the mechanism of gene network regulation at the transcriptional level in *G. bailinae* subjected to extreme temperatures using the RNA-seq technique and combining the growth and physiological performances. The results obtained will not only provide a theoretical basis for understanding the molecular mechanisms of adaptation to high temperature in *G. bailinae*, but also guidance to breed temperature-tolerant seaweed species.

## 2 Materials and methods

### 2.1 Macroalgal samples and temperature treatments

Wild *G. bailinae* specimens were collected from Wushi (N 20°33′, E 109°51′), Guangdong Province, China, in July 2021.The samples were brought back to the lab in a portable fridge at 4°C. Fresh algae were randomly selected and cleaned thoroughly using sterilized seawater. They were then temporarily cultivated in a tank filled with sterile seawater that had been enhanced with f/2 media at 25°C under a 12 L:12 D photoperiod and light intensity of 100 μmol·m^−2^·s^−1^. Three beakers containing 1 L of fresh sterile seawater enriched with f/2 medium were prepared and 3 g of healthy algae was added to each of them. The beakers were then transferred to incubators set at 15°C (LT: low temperature), 25°C (MT: middle temperature), and 35°C (HT: high temperature) for 7 days. Three biological replicates were prepared per treatment and the medium was changed every 2 days. After 7 days of culture, the algae were used for growth and *F*_v_/*F*_m_ measurements, as well as transcriptomic analysis.

### 2.2 Growth and *F*_v_/*F*_m_ measurements

After the treatment, the fresh weight of *G. bailinae* was measured and the relative growth rate (RGR) was determined using the formula supplied by Yong (Yong et al., 2013):


$$RGR\left(\%\cdot d^{-1}\right)=\frac{ln\;M_{7}-ln\;M_{0}}{7}\times 100\%$$

where *M*_0_ represents the starting weight of the algae and *M*_7_ represents their weight after the 7-day treatment.

The maximum photochemical quantum yield of PSII (*F*_v_/*F*_m_) was measured based on Li et al. (Li et al., 2016) after 7 days of treatment at different temperatures using a Hansatech FMS-2 modulated fluorometer (Hansatech Instruments, Norfolk, UK). The formula used was *F*_v_ = *F*_m_ – *F*_0_; the minimum (*F*_0_) and maximum (*F*_m_) fluorescence were obtained by exposing algae that had adapted to darkness for 30 minutes to actinic light and saturated light provided by the fluorometer (Li et al., 2016).

### 2.3 Total RNA extraction, cDNA library construction, and transcriptome sequencing

Total RNA was isolated from *G. bailinae* using the Trizol reagent kit (Invitrogen, Carlsbad, CA, USA) in accordance with the manufacturer’s instructions after 7 days of LT, MT, and HT treatments. An Agilent 2100 Bioanalyzer (Agilent Technologies, Palo Alto, CA, USA) was used to analyze the quality of total RNA and the results were validated *via* RNase-free agarose gel electrophoresis. Following total RNA extraction, Oligo (dT) beads were used to enrich *G. bailinae’s* mRNA. Using random primers, the enriched mRNA was reverse transcribed into cDNA after being broken into small fragments by applying fragmentation buffer. Subsequently, DNA polymerase I, RNase H, dNTP, and buffer were used to create second-strand cDNA. Following purification with Qia Quick PCR extraction kit (Qiagen, Venlo, Netherlands), the cDNA fragments were ligated to the Illumina sequencing adapter after end repair and poly(A) addition. The ligation products were size selected *via* agarose gel electrophoresis, amplified *via* PCR, and then sequenced using Illumina NovaSeq 6000 (Gene Denovo Biotechnology Co., Guangzhou, China).

### 2.4 Analysis of the relationship between samples

Principal component analysis (PCA) was carried out using R (http://www.r-project.org/) on all nine transcriptome datasets to investigate variations in expression patterns between samples. The three duplicates of each temperature treatment were subjected to Pearson correlation analysis in order to evaluate the reproducibility of the transcriptome data.

### 2.5 Filtering of clean reads, *de novo* assembly, and annotation

The raw reads were trimmed by removing those containing adapters, more than 10% of unknown nucleotides, and more than 50% of low quality (Q-value ≤ 20) bases using fastp (version 0.18.0) (Chen et al., 2018). The transcriptome’s *de novo* assembly was carried out using the short-read assembling program Trinity (Grabherr et al., 2011). BLASTx (http://www.ncbi.nlm.nih.gov/BLAST/) with an E-value threshold of 10^5^ was used to compare transcripts to the NCBI non-redundant protein (Nr) database (http://www.ncbi.nlm.nih.gov), the Swiss-Prot protein database (http://www.expasy.ch/sprot), the Kyoto Encyclopedia of Genes and Genomes (KEGG) database (http://www.genome.jp/kegg), and the COG/KOG database (http://www.ncbi.nlm.nih.gov/COG), and annotate the unigenes. Protein function annotations were then obtained based on the best alignment results.

### 2.6 Analysis of differentially expressed genes

The differential expression analysis of RNAs between different groups and between samples was performed using DESeq2 (Love et al., 2014) and edgeR (Robinson et al., 2010) software, respectively. The genes with a parameter of false discovery rate (FDR) below 0.05 and absolute fold change ≥ 3 were considered DEGs.

### 2.7 Verification *via* real-time quantitative PCR

Nine genes associated with temperature changes were randomly selected from the transcriptome results to validate the transcriptome using the 18sRNA (Liu et al., 2021a) gene as a reference gene. The following reaction steps were adopted: 95°C for 300 s, followed by 45 cycles at 95°C for 10 s, 60°C for 15 s, 72°C for 15 s, and reading the fluorescence signal, followed by 1 cycle at 95°C for 10 s, 65°C for 60 s, and 95°C for 1 s. Each reaction was conducted in triplicate. Primer Premier 5.0 (Premier, Toronto, ON, Canada) was used to design each primer. The 2^−ΔΔCt^ (RQ) method was used to determine the relative transcript level (Qin et al., 2021). Primer sequences and qRT-PCR validation results are included in **Supplementary Table S1**.

### 2.8 Statistical analyses

The RGR and *F*_v_/*F*_m_ data were expressed as means ± SD (n ≥ 3). The statistical significance of different treatments was assessed using one-way ANOVA and Duncan’s test in SPSS 25 (IBM Corp., Armonk, NY, USA) with a significance level of *P<* 0.05.

## 3 Results

### 3.1 Growth and *F*_v_/*F*_m_ changes in *G. bailinae* under different temperatures

**Figure 1:**
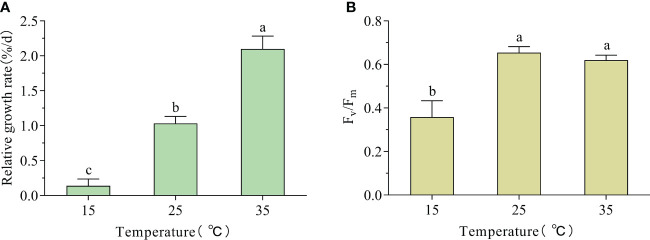
Relative growth rate (RGR) and *F*_v_/F_m_ changes in *G. bailinae* exposed to different temperatures. **(A)** RGR. **(B)** Maximum photochemical quantum yield of PSII (*F*_v_/*F*_m_). Different small letters above the columns indicate a significant difference between different temperature treatments (P< 0.05).

*G. bailinae* maintained a positive growth during the period of culture under the three different temperatures. The HT treatment (35°C) resulted in a maximum growth rate of 2.1%, which was significantly higher than the values obtained at 15°C and 25°C (*P*< 0.01); in particular, it was 15 times higher than that obtained at 15°C (0.14%) (**Figure 1A**). The *F*_v_/*F*_m_ values at 25°C and 35°C were 0.65 and 0.62, respectively, which were significantly higher than the value at 15°C (0.36) (*P<* 0.01) (**Figure 1B**).

### 3.2 Data quality summary

The raw data obtained from the nine samples sequenced were subjected to quality control to obtain clean data (**Supplementary Table S2**). Out of a total of 494,707,094 reads 492,443,386 were retained after removing adapters and low-quality sequences. In each sample, the yield of clean reads was above 99%, the GC content was between 50.94% and 51.56%, and the Q20(%) value was above 98%. The clustering of the assembled transcripts of *G. bailinae* yielded a total of 20,049 single genes (> 200 base pairs) with an N50 count of 4,140 and a length of 1,072 base pairs. The size of single genes ranged from 201 to 8,602 base pairs, with an average length of 769 base pairs and a total length of 15,419,753 base pairs (**Supplementary Table S3**).

### 3.3 Evaluation of the reproducibility of data

The three duplicate samples in each treatment had Pearson correlation coefficients (r) of less than 0.96 (r > 0.80 indicates a strong correlation) (**Supplementary Figure S1A**). PCA showed that the expression patterns of *G. bailinae* differed under the three temperatures (**Supplementary Figure S1B**). Furthermore, qRT-PCR and mRNA-Seq analyses showed that nine randomly selected genes had a similar expression tendency (**Supplementary Table S1**).

### 3.4 Gene function annotation

At present, the whole-genome sequencing of *G. bailinae* has not been completed, therefore, only 20,049 gene transcript sequences were compared to the NCBI non-redundant protein databases Nr, Swiss-Prot, KEGG, and COG/KOG (evalue< 0.00001) using BLASTx. The proteins with the highest sequence similarity to the given unigene and its functional annotation information were obtained. In total, 20,049 genes were assembled from the transcriptome data in this study and 13,095 (65.31%) of them were compared to the four major databases. Of these 20,049 genes, 12,828 (63.98%), 5,246 (26.17%), 5,027 (25.07%), and 9,714 (48.45%) genes matched the Nr, KEGG, KOG, and GO databases, respectively.

In the Nr database, the main match obtained was *Gracilariopsis chorda* (84.82%), which belongs to the same genus as *G. bailinae*, in line with the taxonomic status distinction (**Supplementary Figure S2**). Cellular processes, environmental information processing, genetic information processing, metabolism, and organic systems were the five primary functional categories of the genes that matched the KEGG database. Each category was subdivided into secondary groups of functions. In particular, the largest number of unigenes were involved in “global and overview maps” (1,445), followed by “translation” (750) and “folding, sorting, and degradation” (409) (**Supplementary Figure S3**). The genes that matched the KOG database were divided into 25 groups: the largest was “translation, ribosomal structure and biogenesis” (781), followed by “general function prediction only” (701), and the smallest was “cell motility” (5) (**Supplementary Figure S4**). A total of 5,824 (29.05%) genes were matched to Swiss-Prot database. The number of genes matched in all databases was 4611 (23.00%) (**Supplementary Figure S5**).

Based on the Nr annotation information, Blast2GO was used to obtain the GO function annotations (Ashburner et al., 2000; Conesa et al., 2005). Three ontologies in GO were used to categorize the genes’ molecular functions (MF), cellular components (CC), and biological processes (BP). The 9,714 unigenes annotated into the GO database by GO annotation were classified into 50 subclasses, accounting for 48.45% of all unigenes, and the same unigenes could be annotated to different subclasses. The top three subclasses of the GO ontologies were: cellular process, metabolic process, and localization with 6,637, 5,845, and 1,497, genes, respectively, for BP; cellular anatomical entity, protein-containing complex, and virion component, with 4,253, 1,958 and 110 genes, respectively, for CC; and catalytic activity, binding, and transporter activity, with 5,066, 4,771, and 802 genes, respectively, for MF (**Supplementary Figure S6**).

### 3.5 Analysis of DEGs

#### 3.5.1 Overview of DEGs under stress

**Figure 2:**
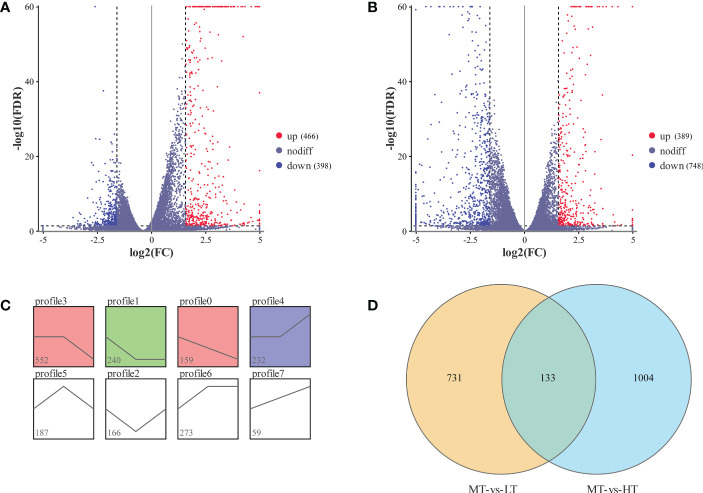
Overview of DEGs in ***G***. *bailinae* exposed to different temperatures. **(A)** Volcano plots of the DEGs in MT *vs* LT. **(B)** Volcano plots of the DEGs in MT *vs* HT. **(C)** Trend analysis of DEGs in MT *vs* LT and MT *vs* HT. Significant enrichment trends are shown by colored blocks (*P* ***<*** 0.05). The bottom-left number represents the number of genes. **(D)** Venn diagram depicting the number of DEGs obtained in the MT *vs* LT and MT *vs* HT comparisons.

Two comparison groups, MT *vs* LT and MT *vs* HT, were established to investigate the variations in gene expression under different temperature treatments using log2(FC) ≥ 1.5 and FDR ≤ 0.05 as screening conditions. A total of 1,868 genes differentially expressed under different temperatures were identified: 1137 DEGs (389 upregulated and 748 downregulated) and 864 DEGs (466 up-regulated and 398 down-regulated) were identified in MT *vs* HT and MT *vs* LT, respectively (**Figures 2A, B**); 133 DEGs were co-regulated under LT and HT treatments (**Figure 2D**). In addition, to explore the gene expression patterns of *G. bailinae* under varying temperatures, a trend analysis of DEGs was performed for MT *vs* LT, and MT *vs* HT. All DEGs were clustered into eight trend profiles, and 1183 DEGs were significantly enriched in four of them, i.e., 3, 1, 0, and 7 (*P*< 0.05), which is shown in colored blocks in **Figure 2C**. Three of the four significantly enriched trends, i.e., about 80% of the significantly enriched DEGs in the trend analysis, showed a decrease with increasing temperature, while only one trend showed an increase with temperature.

#### 3.5.2 GO classification and KEGG pathway analysis of DEGs in MT *vs* LT and MT *vs* HT

GO and KEGG analyses were conducted to examine the functions of DEGs. The GO classification results showed that DEGs were involved in three groups of functions, i.e., biological processes, molecular functions, and cellular components, and most genes were specifically classified as being involved in cellular processes (LT: 275, HT: 388), metabolic processes (LT: 241, HT: 344), and catalytic activity (LT: 200, HT: 311) both in MT *vs* LT and MT *vs* HT (**Supplementary Figure S7**). KEGG enrichment analysis indicated that DEGs were mainly enriched in four KEGG B classes: carbohydrate metabolism, energy metabolism, global and overview maps, and lipid metabolism (**Supplementary Figure S8** and **Table 1**).

**Table 1:** Significantly enriched KEGG pathways in MT *vs* HT and MT *vs* LT.

Pathway ID	Pathway	KEGG_B_class	No. of DEGs	Pvalue
MT *vs* LT				
ko01200	Carbon metabolism	Global and overview maps	20	7.28E-04
ko00196	Photosynthesis - antenna proteins	Energy metabolism	7	1.07E-03
ko00520	Amino sugar and nucleotide sugar metabolism	Carbohydrate metabolism	7	1.25E-03
ko01040	Biosynthesis of unsaturated fatty acids	Lipid metabolism	4	3.50E-03
ko01100	Metabolic pathways	Global and overview maps	74	3.71E-03
ko00785	Lipoic acid metabolism	Metabolism of cofactors and vitamins	2	9.72E-03
ko01110	Biosynthesis of secondary metabolites	Global and overview maps	40	1.32E-02
ko02010	ABC transporters	Membrane transport	4	2.37E-02
ko00051	Fructose and mannose metabolism	Carbohydrate metabolism	5	2.83E-02
ko00592	alpha-Linolenic acid metabolism	Lipid metabolism	2	3.13E-02
ko00260	Glycine, serine and threonine metabolism	Amino acid metabolism	6	3.39E-02
ko00710	Carbon fixation in photosynthetic organisms	Energy metabolism	7	3.43E-02
ko00630	Glyoxylate and dicarboxylate metabolism	Carbohydrate metabolism	7	3.89E-02
ko00562	Inositol phosphate metabolism	Carbohydrate metabolism	4	4.58E-02
MT *vs* HT				
ko01100	Metabolic pathways	Global and overview maps	124	3.93E-06
ko00195	Photosynthesis	Energy metabolism	17	1.22E-05
ko03010	Ribosome	Translation	43	4.28E-04
ko00450	Selenocompound metabolism	Metabolism of other amino acids	7	8.26E-04
ko00710	Carbon fixation in photosynthetic organisms	Energy metabolism	13	9.64E-04
ko04712	Circadian rhythm - plant	Environmental adaptation	3	1.75E-02
ko00051	Fructose and mannose metabolism	Carbohydrate metabolism	7	1.79E-02
ko01200	Carbon metabolism	Global and overview maps	23	1.89E-02
ko00030	Pentose phosphate pathway	Carbohydrate metabolism	8	2.06E-02
ko00010	Glycolysis/Gluconeogenesis	Carbohydrate metabolism	13	2.28E-02
ko00190	Oxidative phosphorylation	Energy metabolism	17	2.77E-02
ko00196	Photosynthesis - antenna proteins	Energy metabolism	6	4.39E-02

#### 3.5.3 Effect of different temperatures on specific metabolic pathways

Based on the DEG analysis in KEGG, the following nine pathways related to the energy, carbohydrate, and lipid metabolisms were selected to investigate the specific effects of high and low temperatures on *G. bailinae*: photosynthesis (ko00195), photosynthesis-antenna proteins (ko00196), carbon fixation in photosynthetic organisms (ko00710), glycolysis/gluconeogenesis (ko00010), pentose phosphate pathway (PPP) (ko00030), pyruvate metabolism (ko00620), citrate cycle (TCA cycle) (ko00020), biosynthesis of unsaturated fatty acids (ko01040), and alpha-linolenic acid metabolism (ko00592).

##### 3.5.3.1 Pathways related to photosynthesis

**Figure 3:**
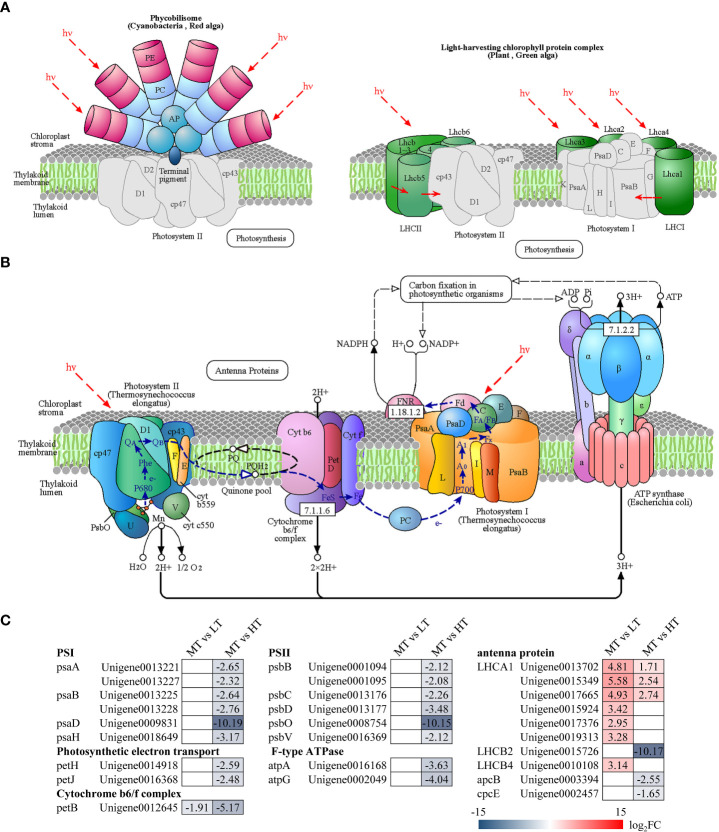
Pathways of photosynthesis and photosynthetic antenna proteins: **(A)** photosynthetic antenna protein pathway (ko00196), **(B)** photosynthesis pathway (ko00195), **(C)** DEGs involved in photosynthesis and photosynthetic antenna proteins. The number in each cell is the log2 fold change (log2FC). Red and blue gradients indicate the upregulation and downregulation of unigenes, respectively, while the white color represents log2FC**<** 1.5 or FDR > 0.05. The diagrams of photosynthesis and photosynthetic antenna proteins were obtained from the KEGG website.

Comparative transcriptome analysis identified 17 DEGs involved in photosynthesis, which participated in the formation of PS II (psaA, psaB, psaD, psaH), PS I (psbB, psbC, psbD, psbO, psbV), the cytochrome b6/f complex (petB), F-type ATPase (atpA, atpG), and photosynthetic electron transport (petH, petJ) (**Figure 3**). All these DEGs were significantly downregulated in MT *vs* HT, while only one significantly downregulated gene (petB) was enriched in MT *vs* LT. In addition, the analysis identified 10 DEGs associated with photosynthetic antenna proteins and involved in the synthesis of allophycocyanin beta subunit (apcB), phycocyanobilin lyase subunit alpha (cpcE), and light-harvesting complex I chlorophyll a/b binding proteins 1, 2, and 4 (LHCA1, LHCB2, and LHCB4). Most of these DEGs were upregulated in LT and HT treatments, and only three related to LHCB2, apcB, and cpcE were significantly downregulated in MT *vs* HT.

##### 3.5.3.2 Pathways related to carbohydrate and energy metabolism

**Figure 4:**
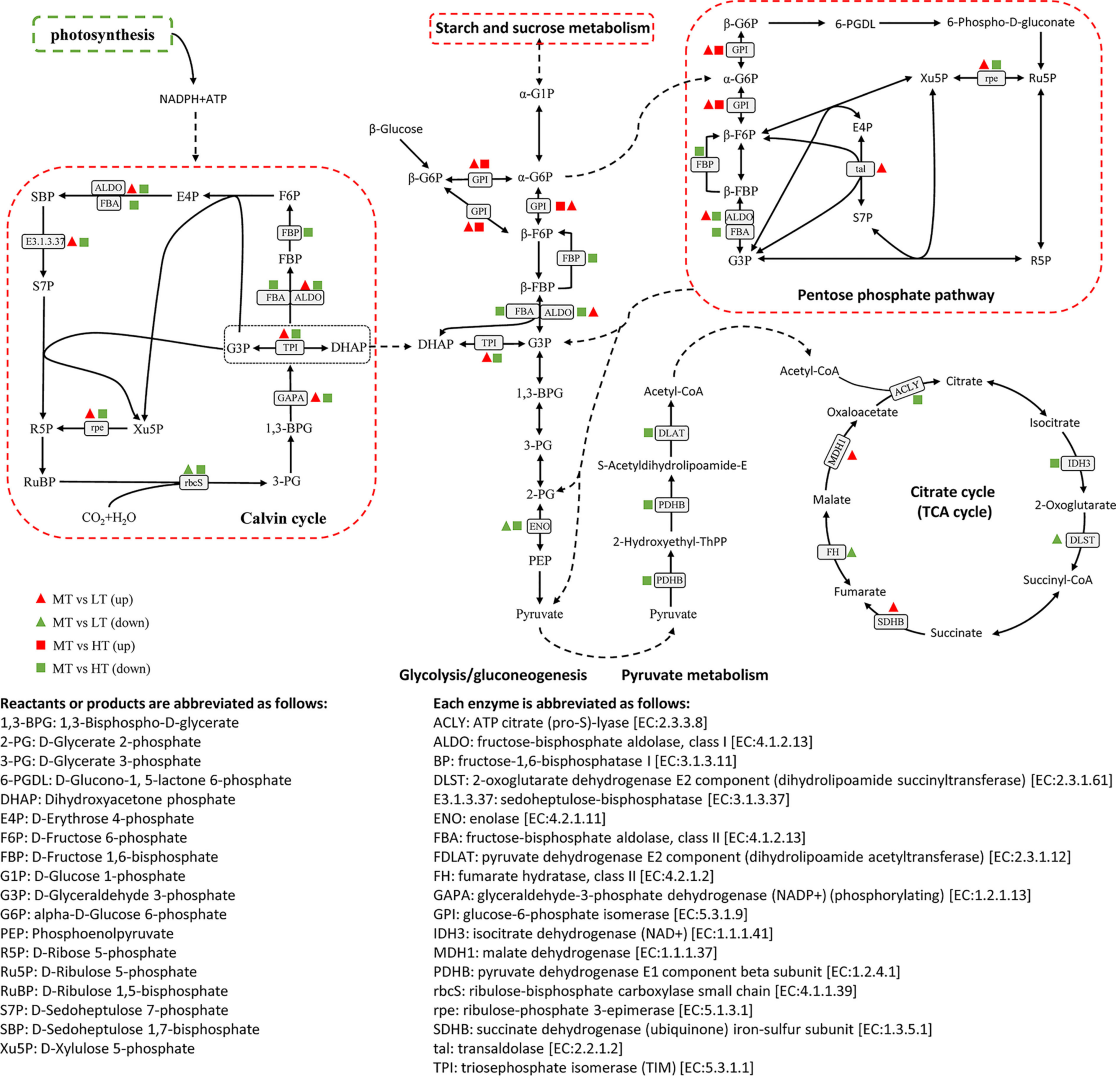
Pathways of carbon fixation in photosynthetic organisms, glycolysis/gluconeogenesis, pyruvate metabolism, pentose phosphate, and citrate cycle (TCA cycle). Detailed descriptions of the DEGs are included in **Supplementary Table S4**.

Transcriptome analysis showed that DEGs were significantly enriched in the pathways related to carbohydrate and energy metabolisms and played a role in the response to temperature changes in *G. bailinae*. The NADPH and ATP produced by photosynthesis can be used for carbon dioxide fixation (**Figure 3B**). A total of 11 DEGs were significantly enriched in the pathway of carbon fixation in photosynthetic organisms (Calvin cycle), encoding ALDO, E3.1.3.37, rbcS, FBP, FBA, rpe, TPI, and GAPA. Most of the genes involved in this pathway were significantly downregulated under the HT treatment, while the opposite was observed under the LT treatment (**Figure 4**). In addition, it is worth noting that the gene encoding rbcS was significantly downregulated under both treatments, which may be associated with different temperature response mechanisms.

The intermediate products synthesized by the Calvin cycle, i.e., propyl phosphates (glyceraldehyde phosphate and dihydroxyacetone phosphate), can be further used in the glycolysis/glycogenesis pathway to synthesize sucrose and starch, enter the TCA cycle to produce energy, or flow to PPP to produce NADPH. In MT *vs* HT, 15 DEGs were significantly enriched in the above pathways, encoding ALDO, FBA, TPI, FBP, GPI, ENO, IDH3, ACLY, rpe, PDHB, and DLAT. Most of the DEGs were downregulated under the HT treatment, and only one DEG encoding GPI was upregulated (**Figure 4**). In MT *vs* LT, a total of 10 DEGs were significantly enriched in the glycolysis/gluconeogenesis, PPP, TCA cycle, and pyruvate metabolism pathways, encoding ALDO, TPI, GPI, ENO, MDH1, SDHB, DLST, FH, rpe, and tal. Specifically, the DEGs encoding ENO, DLST, and FH were significantly downregulated, while the remaining seven DEGs were significantly upregulated.

##### 3.5.3.3 Analysis of pathways related to lipid metabolism

**Figure 5:**
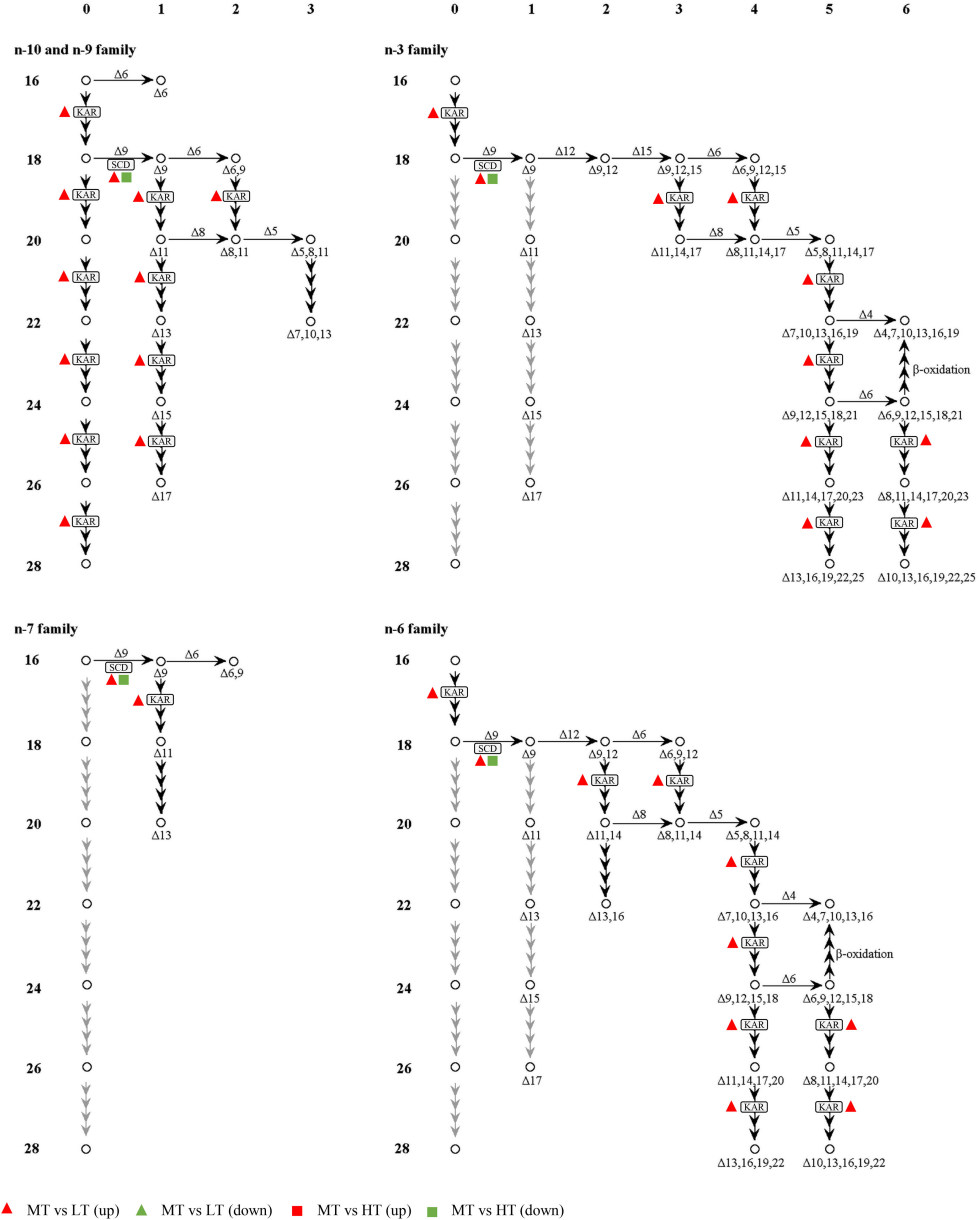
Pathways of biosynthesis of unsaturated fatty acids. The left side and top of the figure show the number of carbon atoms (16~28), and number of double bonds (1~6), respectively. The detailed descriptions of the DEGs are included in **Supplementary Table S4**.

Transcriptome analysis revealed that temperature induced the differential expression of genes related to lipid metabolism, especially those involved in the biosynthesis of unsaturated fatty acids and alpha-linolenic acid metabolism. Four DEGs encoding desaturation-related enzymes (SCD) and chain extension-related enzymes (KAR) in the biosynthesis of unsaturated fatty acids were significantly differentially expressed in different comparison groups. Both SCD and KAR were upregulated under the LT treatment, while only SCD was downregulated under the HT treatment (**Figure 5**).

**Figure 6:**
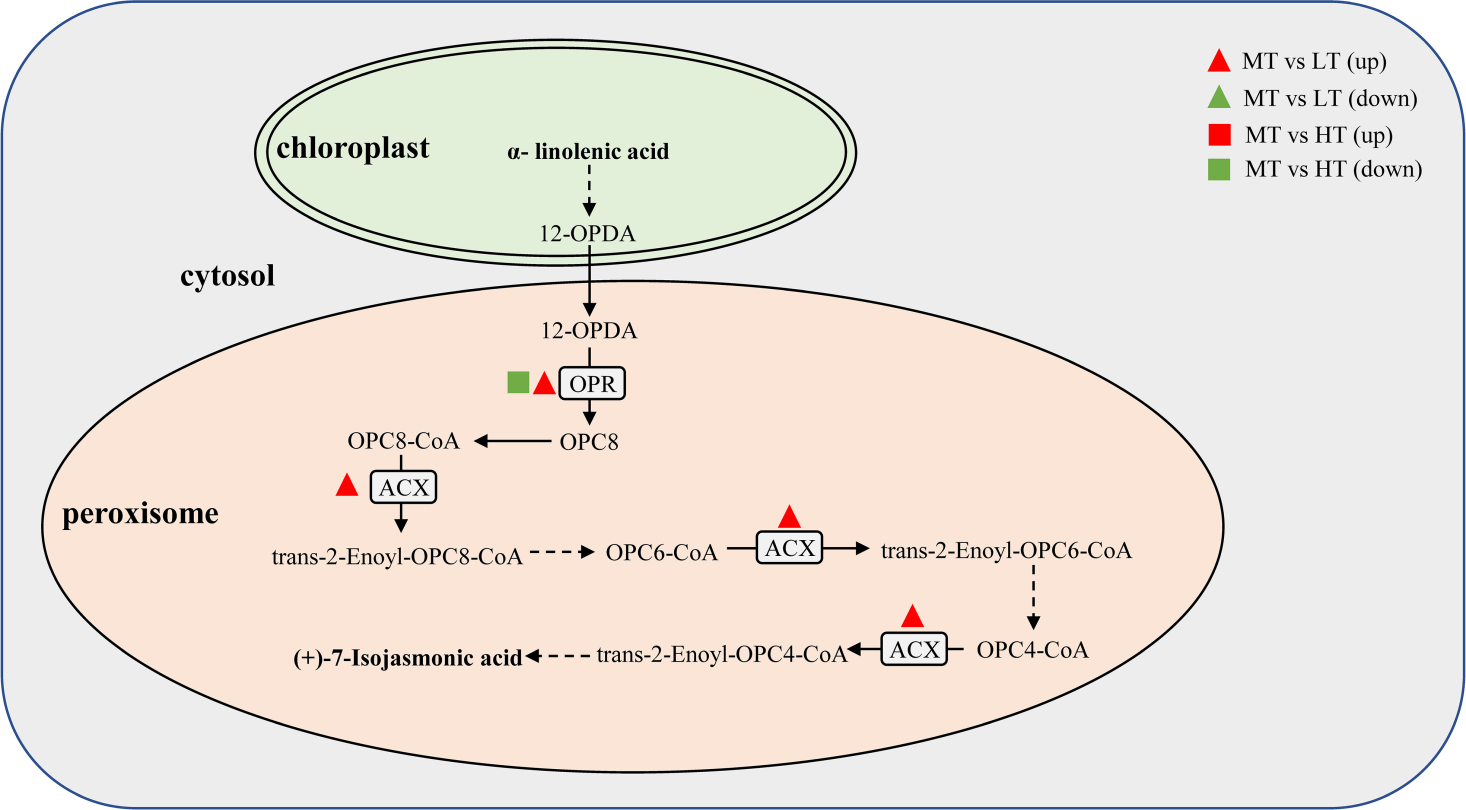
Pathways of the alpha-linolenic acid metabolism. The solid and dashed arrows indicate one-step and multi-step processes, respectively. The detailed descriptions of the DEGs are included in **Supplementary Table S4**.

**Figure 6** shows the effect of temperature on the metabolism of alpha-linolenic acid in *G. bailinae*. Phosphatidylcholine in the plastid membrane is oxidized by lipase to form alpha-linolenic acid, which then undergoes a series of oxidations to form oxo-phytodienoic acid (OPDA) in the plastid. Then, OPDA enters the peroxisomes through the ABC transporter and forms (+)-7-iso-jasmonic acid after reduction and 3-step β-oxidation. In the present study, three DEGs encoding acyl-CoA oxidase (ACX) and 12-oxophytodienoic acid reductase (SCD) were significantly activated after the LT and HT treatments. Under HT, only SCD was significantly downregulated, while under LT both ACX and SCD were significantly upregulated.

## 4 Discussion

As ocean warming continues to increase, there are inevitable consequences for the survival of macroalgae; however, species that can adapt to heat stress are better prepared to cope with the warmer conditions (Ji and Gao, 2021; Association, 2022). The present study found that the optimum growth state of *G. bailinae* was at 35°C. Based on this observation, transcriptomic analysis was first conducted to reveal the mechanism behind the adaptability of this macroalgae to high temperatures. The results of RNA-seq analysis showed that the process is associated with the regulation of the energy, carbohydrate, and lipid metabolism.

### 4.1 Growth and *F*_v_/*F*_m_ response to temperature changes

Macroalgae are expected to be especially sensitive to ocean warming since the rates of their biochemical and physiological processes are substantially regulated by ambient temperature (as observed in ectotherms) (Davison, 1991; Brown et al., 2004; Graba-Landry et al., 2018). Typically, within a species’ thermal tolerance range, the rates of physiological processes and other parameters (e.g., growth) increase with temperature until they reach a thermal optimum and rapidly decline thereafter (Brown et al., 2004; Graba-Landry et al., 2018). Different algae show different adaptations to temperature variation, and growth rate is an important indicator that visually reflects their growth status (Yang et al., 2021; Gao et al., 2022): the highest growth rate is recorded at the optimum growth temperature. For example, the highest growth rate of *G. lemaneiformis* (Liu et al., 2017), *Ulva prolifera* (Cui et al., 2015), and *Caulerpa sertularioides* (Zhong et al., 2021) was observed at 23°C, 20°C, and 26°C, respectively. In the present study, the RGR of *G. bailinae* reached its maximum at 35°C and growth was significantly inhibited under the lower temperatures tested. *F*_v_/*F*_m_ is the maximum photochemical quantum yield of PSII, which reflects the potential maximum photosynthetic capacity of macroalgae and can also indirectly reflect their growth state and living environment (Goltsev et al., 2016; Cui et al., 2019; Zhong et al., 2021). Studies have indicated that *F*_v_/*F*_m_ is a heat-sensitive indicator, which makes it an important tool for the evaluation of plants’ heat tolerance (Yamada et al., 1996; Wahid et al., 2007; Wu et al., 2019). For example, in *G. lemaneiformis*, this parameter was shown to decrease under unsuitable temperatures (Liu et al., 2017). A notable drop in *F*_v_/*F*_m_ was also observed in *Saccharina japonica* under high temperature stress, which was caused by the deactivation of PSII (Shindo et al., 2022). In the present study, the analysis of *F*_v_/*F*_m_ in *G. bailinae* indicated that 25°C and 35°C were suitable temperatures, but the parameter decreased significantly at 15°C. Obviously, the changes in RGR and *F*_v_/*F*_m_ under different temperatures indicate that *G. bailinae* has a remarkable capacity to adapt to high temperatures.

### 4.2 Effect of temperature on photosynthesis

It has long been acknowledged that plant photosynthesis is one of the most temperature-sensitive processes (Li et al., 2019). This process depends on electron transport into the photosynthetic system and it gives algae the energy they need to grow and develop (Qin et al., 2021). In the cells of red algae, light energy absorbed by the phycobillisome and the light-harvesting chlorophyll protein complex (LHC) drives the flow of electrons from H_2_O to NADP^+^, which promotes the reduction of NADP^+^ to NADPH (Croce and Van Amerongen, 2014). At the same time, ATP synthase uses the electrochemical gradient generated simultaneously across the thylakoid membrane for ATP synthesis (Croce and Van Amerongen, 2014). NADPH and ATP represent the basis for all subsequent chemical reactions (Croce and Van Amerongen, 2014). Algae can regulate the above processes by controlling gene expression to resist or adapt to environmental changes, that is, regulating the synthesis of NADPH and ATP. Wang et al. (2018) reported that the protein-related genes of the photosynthetic antenna in wild-type *P. haitanensis* were significantly downregulated under high temperature stress, impeding the absorption of sufficient light to activate photosynthesis, and therefore resulting in insufficient energy to resist heat stress (Wang et al., 2018). Another study on *Amphiroa fragilissima* revealed that the expression levels of several DEGs involved in photosynthesis were suppressed under high temperature stress compared to those in the control group (Yang et al., 2021). However, in the present study, the photosynthetic activity of *G. bailinae* remained normal despite the downregulation of photosynthesis-related genes, which was not in line with the results of the above-mentioned studies. The downregulation of genes in most heat-sensitive algae under temperature stress may lead to fatal results, but *G. bailinae* has a high temperature adaptability, and 35°C is its suitable growth temperature. A study on the high-temperature tolerant *P. haitanensis* showed that this species can reduce unnecessary energy consumption by decreasing the rate of photosynthesis and energy metabolism (Wenlei et al., 2018). A proteomic analysis similarly indicated that the *P. haitanensis* strain Z-61 resists high temperature stress by downregulating photosynthesis (Xu et al., 2014). Similarly, *G. bailinae* regulates NADPH and ATP synthesis by downregulating photosynthesis-related genes to decrease the electron transfer rate and H^+^ production in the photosynthetic system, thereby potentially reducing unnecessary energy consumption and ultimately adapting to high temperature environments. In addition, as observed for the compensatory reaction of gene expression (Dal Bosco et al., 2004), the high expression of LHCA1-related genes in photosynthetic antenna proteins may compensate for the decreased light capture efficiency caused by the downregulation of genes related to LHCB2, apcB, and cpcE, so as to meet the light energy demand of *G. bailinae* and achieve a higher growth rate.

### 4.3 Effect of temperature on carbohydrate and energy metabolisms

In plants, a metabolic regulatory network is frequently engaged in the response to abiotic stimuli (Wenlei et al., 2018). Previous studies have shown that *P. haitanensis* under stress can reduce unnecessary energy consumption to prevent cell damage (Xu et al., 2014; Xu et al., 2016). However, when stress levels become excessive, the corresponding energy shortage in algae would result in an enhancement of the inherent pathways of carbohydrate metabolism and the induction of alternative pathways, such as glycolysis, to provide energy and the related carbon skeletons for key metabolic processes (Obata and Fernie, 2012; Wenlei et al., 2018). Wang et al. (Wang et al., 2018) corroborated this claim by studying the heat-tolerant *P. haitanensis*, whose genes showed a tendency to be upregulated under high temperature stress to maintain the energy and associated carbon skeleton for key metabolic processes. In the present study, a joint analysis was performed for the following five pathways related to the carbohydrate and energy metabolisms: Calvin cycle, glycolysis/gluconeogenesis, PPP, pyruvate metabolism, and TCA cycle. In our study, we found that only the DEG encoding GPI was significantly upregulated under HT treatment, while other DEGs enriched in these pathways were downregulated (**Figure 4**). The accumulation of sugar phosphates associated with glycolysis and the oxidative pentose phosphate pathway (OPPP) indicated that glycolytic carbon flow may be rerouted into the OPPP to produce NADPH for antioxidative action (Obata and Fernie, 2012; Wenlei et al., 2018). GPI catalyzes the interconversion between F6P and G6P. Further oxidation of G6P in the PPP can produce a large amount of NADPH, which may be one of the remedies for the decrease in NADPH synthesis during photosynthesis. The Calvin cycle, which comprises 13 chemical steps and 11 enzymes that catalyze them, is the main mechanism for carbon fixation in C3 plants (Tamoi et al., 2005). The cycle converts atmospheric CO_2_ into carbon skeletons that are utilized to produce starch and sucrose using the byproducts of the light reactions of photosynthesis (i.e., ATP and NADPH) (Raines, 2003). Gene downregulation during the Calvin cycle is generally thought to lead to a reduced synthesis of related enzyme proteins, especially the key protein Rubisco. However, in this study, the expression of genes related to the Rubisco small subunit rbcS was downregulated under HT treatment, but the biomass of *G. bailinae* was relatively high. Related research shows that Rubisco activase induced the activation of Rubisco, but the synthesis of this protein (transcription and translation) and enzyme had different sensitivities to long-term heat stress (Almeselmani et al., 2012). Therefore, due to the adaptability of *G. bailinae* to high temperatures, Rubisco activase may activate Rubisco more obviously under high temperature conditions, thereby improving the carbon sequestration efficiency of the protein and reducing its absolute content during the Calvin cycle. However, further studies are needed to confirm this. The TCA cycle promotes the oxidation of respiratory substrates for ATP generation, and therefore plays a crucial role in plant resistance to abiotic challenges (Sweetlove et al., 2010; Obata and Fernie, 2012; Wenlei et al., 2018). It is worth noting that the genes encoding ACLY and IDH3 were significantly downregulated in the TCA cycle. IDH3 catalyzes the conversion of isocitrate to alpha-ketoglutarate to produce NADH. The downregulation of IDH3 indicated that *G. bailinae* adapts to high temperatures by reducing unnecessary energy consumption. In addition, the downregulation of other genes in the Calvin cycle, glycolysis/gluconeogenesis, and pyruvate metabolism pathways also indicated that *G. bailinae* slows the rate of energy or carbohydrate metabolisms to adapt to high temperatures. On the contrary, under low temperature stress, due to energy shortage, this macroalgae needs to produce more energy by overexpressing genes associated with the synthesis of energy molecules, such as MDH1 and SDHB, to maintain the key life activities of cells.

### 4.4 Effect of temperature on lipid metabolism

In response to temperature stress, plants typically control the percentage of polyunsaturated fatty acids to alter membrane fluidity (Sun et al., 2015). Two aspects are involved in the production of unsaturated fatty acids: fatty acid peptide chain extension and desaturation (Qin et al., 2021). Fatty acids, which are the major components of membrane phospholipids, induce desaturation to promote cell membrane fluidity and stability, hence minimizing cellular damage (Qin et al., 2021). Transcriptome analysis of *G. lemaneiformis* revealed that the expression levels of genes associated with fatty acid extension and desaturation under normal temperatures were significantly lower than those under low temperature stress (Qin et al., 2021). Wang et al. (2018) also pointed out that genes related to unsaturated fatty acid synthesis in *P. haitanensis* were more significantly downregulated under normal temperatures than under high temperature stress. In this study, the genes associated with desaturation (SCD) were significantly downregulated under the HT treatment, while those associated with both fatty acid chain extension (KAR) and desaturation (SCD) were significantly upregulated under the LT treatment. This indicates that *G. bailinae* may regulate the membrane’s lipid fluidity to adapt to high temperatures by gradually downregulating the genes related to the biosynthesis of unsaturated fatty acids.

Alpha-linolenic acid (a C18 polyunsaturated acid) is a biosynthetic substrate for jasmonic acid (JA) and its diverse derivatives, which are lipid-derived signaling molecules involved in the regulation of numerous plant processes, including growth, reproductive development, photosynthesis, and responses to abiotic and biotic stresses (Bhatla, 2018). In plants, alpha-linolenic acid is perhaps the most prevalent and chemically diverse source of polyunsaturated fatty acids (Gerwick et al., 1991; Leung et al., 2022). JA can further form methyl jasmonate, which is considered to be crucial for algae’s ability to withstand heat (Qin et al., 2021). Exogenous methyl jasmonate has been found to reduce the negative effects of high temperature stress on a number of pathways in *G. lemaneiformis* (Wang et al., 2017). A transcriptome analysis of this species under low temperature stress also revealed that the activation of crucial genes associated with methyl jasmonate production was dramatically elevated (Qin et al., 2021). Su et al. (Su et al., 2019) reported that heat stress induced the upregulation of genes involved in the metabolism of alpha-linolenic acid in wheat. In the present study, it was revealed that the genes encoding OPR (the enzyme 12-oxophytodienoic acid reductase that catalyzes the conversion of 12-OPDA to OPC8) were significantly downregulated under the HT treatment. In contrast, those encoding OPR and ACX (acyl-CoA oxidase, which plays a crucial role in the three rounds of β-oxidation for the synthesis of JA acid from alpha-linolenic acid (Liu et al., 2021b)) were significantly upregulated in the LT treatment (**Figure 6**). These results suggest that *G. bailinae* can adapt to high temperatures by gradually limiting JA synthesis.

## 5 Conclusions

In summary, this study proved that the appropriate growth temperature for *G. bailinae* is 25–35°C, and the optimum is 35°C. It also revealed that this species modifies nine metabolic pathways related to the energy, carbohydrate, and lipid metabolisms to adapt to high temperatures. *G. bailinae* regulates the synthesis of NADPH and ATP by downregulating genes related to pathways of the energy and carbohydrate metabolisms to reduce unnecessary energy consumption. In addition, it controls the synthesis of unsaturated fatty acids and JA by downregulating the genes related to lipid metabolism to regulate the fluidity of membrane lipids and the content of lipid-derived signaling molecules, respectively. These results provide us with a better understanding of the molecular mechanisms underlying the adaptation of *G. bailinae* to high temperatures, which may assist in the development of technologies and breeding strategies to improve the heat tolerance of *Gracilaria* spp.

## Data availability statement

The datasets presented in this study can be found in online repositories. The names of the repository/repositories and accession number(s) can be found below: BioProject, PRJNA925653.

## Author contributions

YH, JC and EX conceived and designed this study. YH conducted the experiments, analyzed the results, and wrote the original manuscript. EX is responsible for obtaining research funding. JC revised and improved the original manuscript. SW and XC conducted a preliminary analysis of the data. JL, YG, RX and BH assisted the completion of the experiment. All authors discussed the results at various stages. All authors contributed to the article and approved the submitted version.
