# Supplementary material for: Transcriptome analysis reveals the molecular mechanisms of adaptation to high temperatures in Gracilaria bailinae
Source: Front Plant Sci. 2023 Apr 14;14:1125324. doi: 10.3389/fpls.2023.1125324 (PMC10140531; doi:10.3389/fpls.2023.1125324)
Supplement: Supplementary file 1 [file DataSheet_1.pdf]

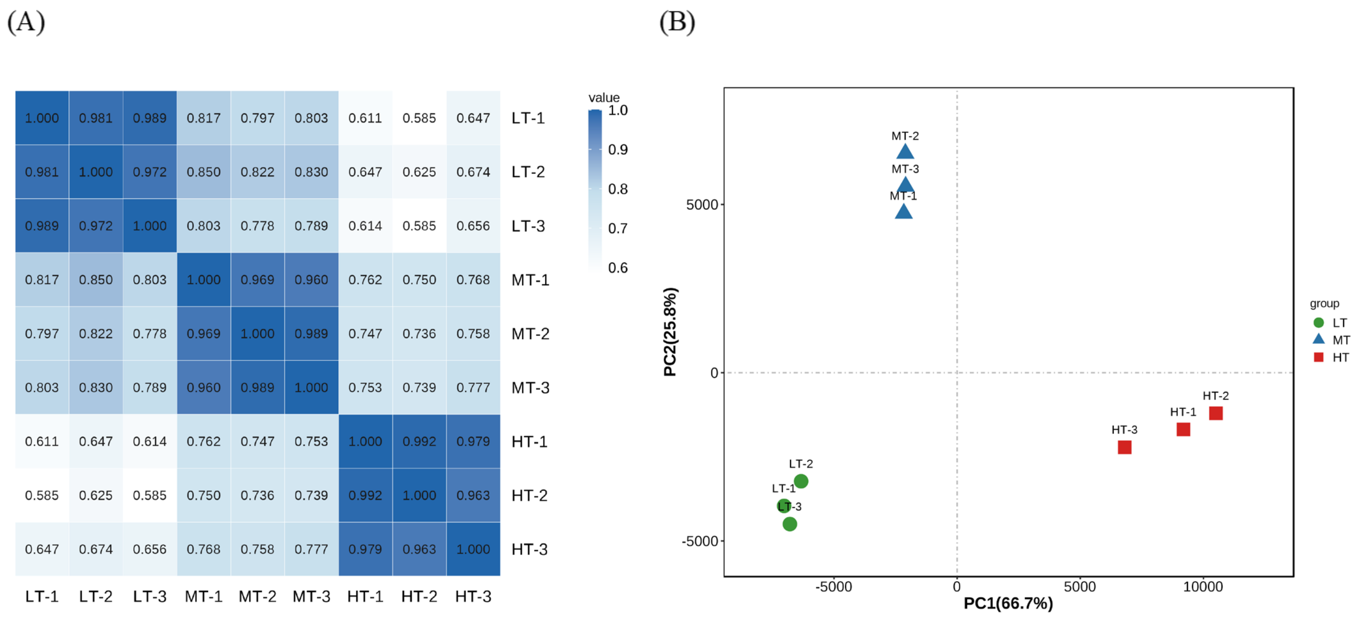

Figure S1. Sample correlation analysis. (A) Heat map of Pearson correlation coefficients. (B) Two-dimensional diagram of principal component analysis.

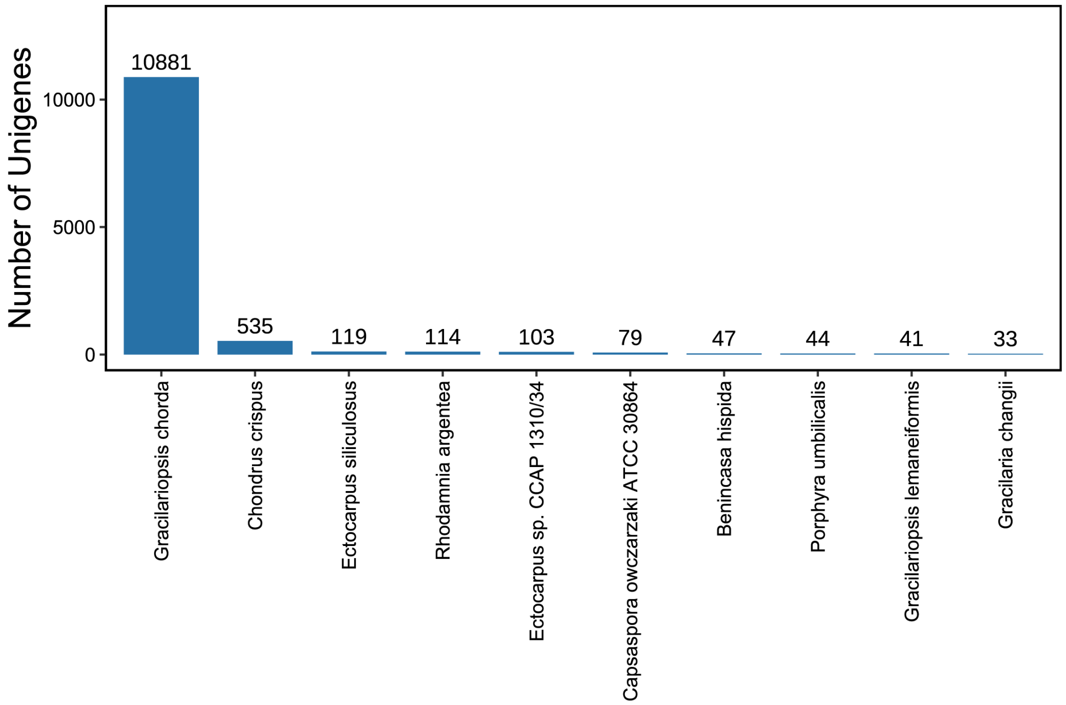

Figure S2. Statistics of the number of species compared from the Nr database based on the transcriptome results of *G. bailinae*.

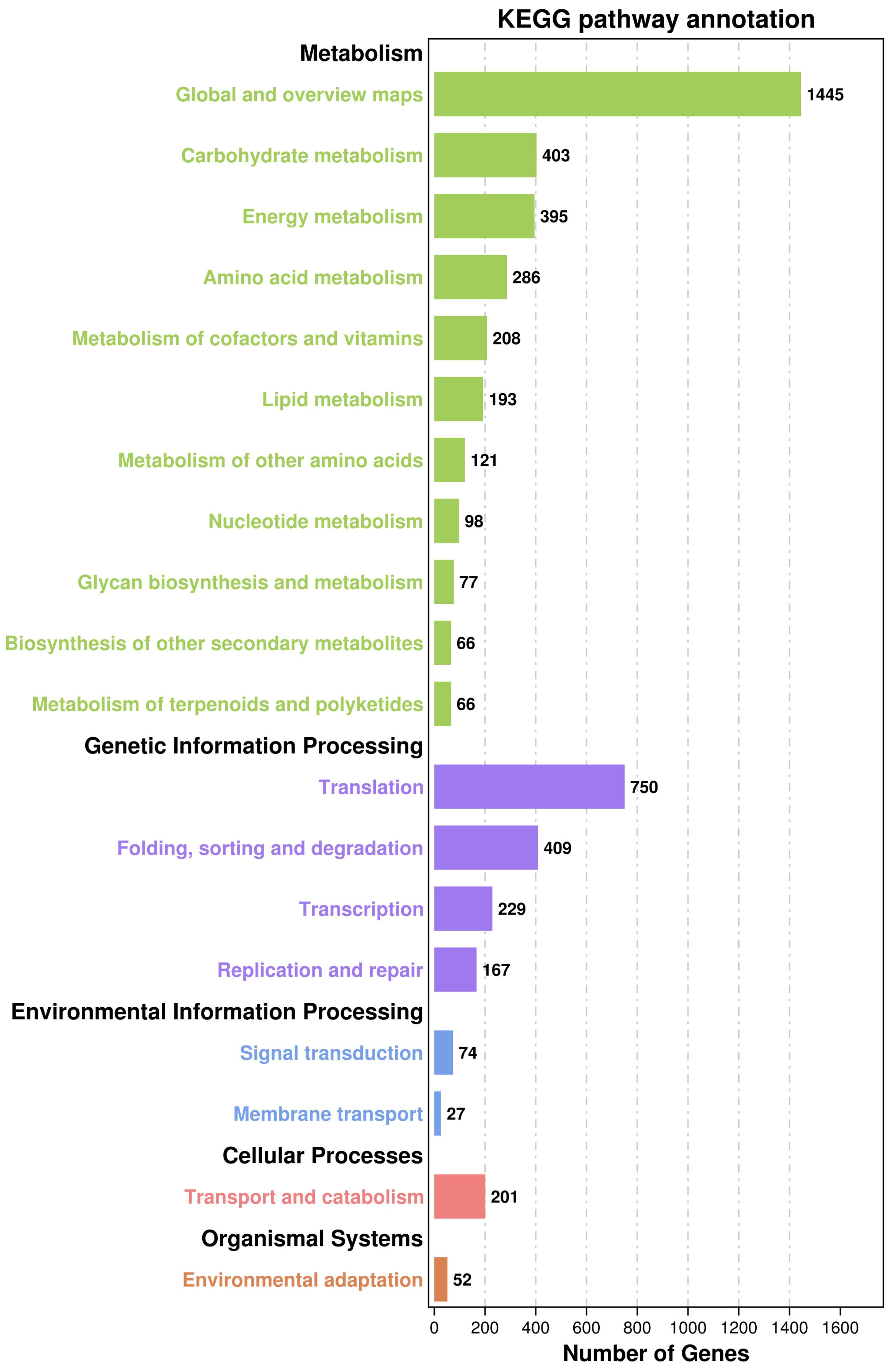

Figure S3. Statistics of Pathway results annotated from the KEGG database based on the transcriptome results of *G. bailinae*.

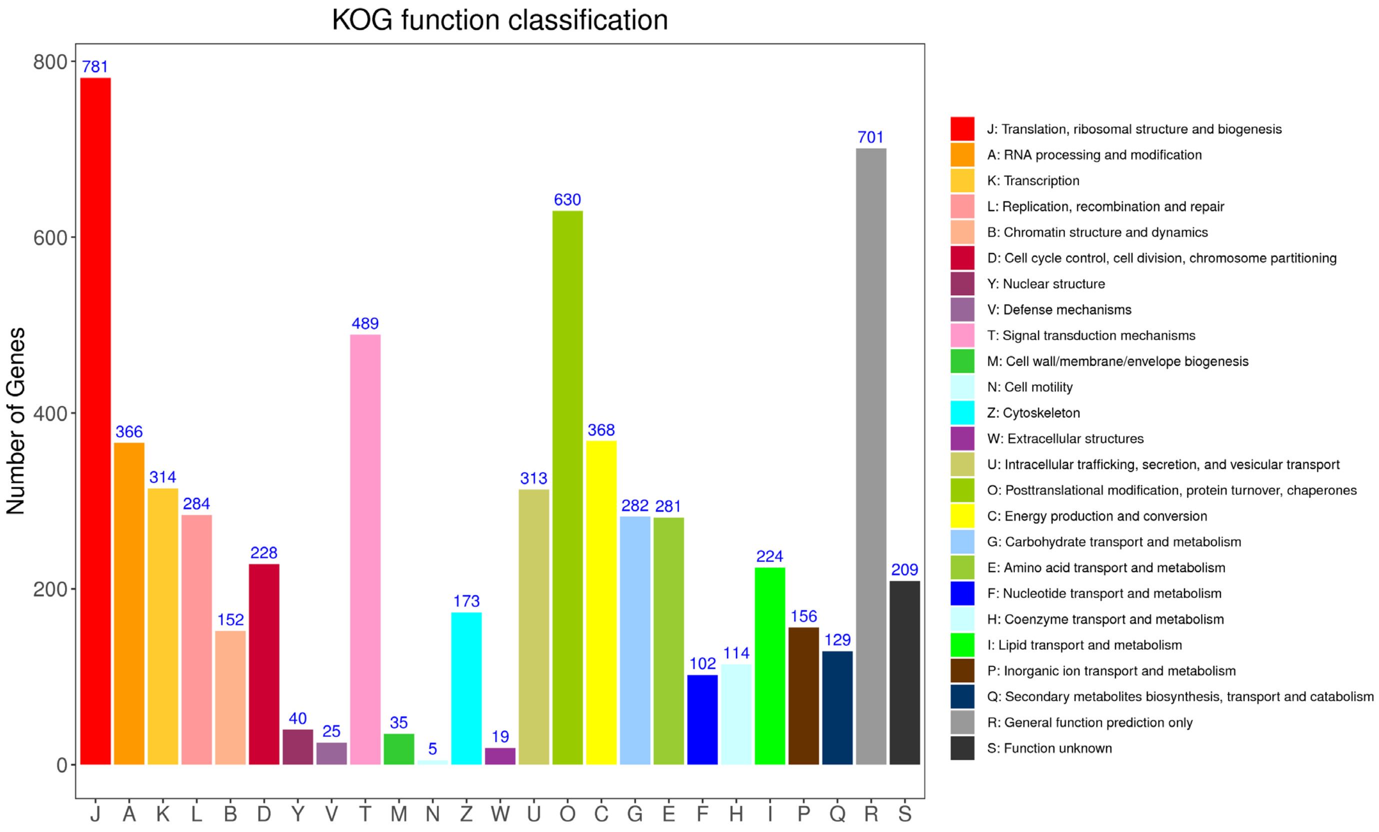

Figure S4. Statistics of KOG functional classification results annotated from the COG/KOG database based on the transcriptome results of *G. bailinae*.

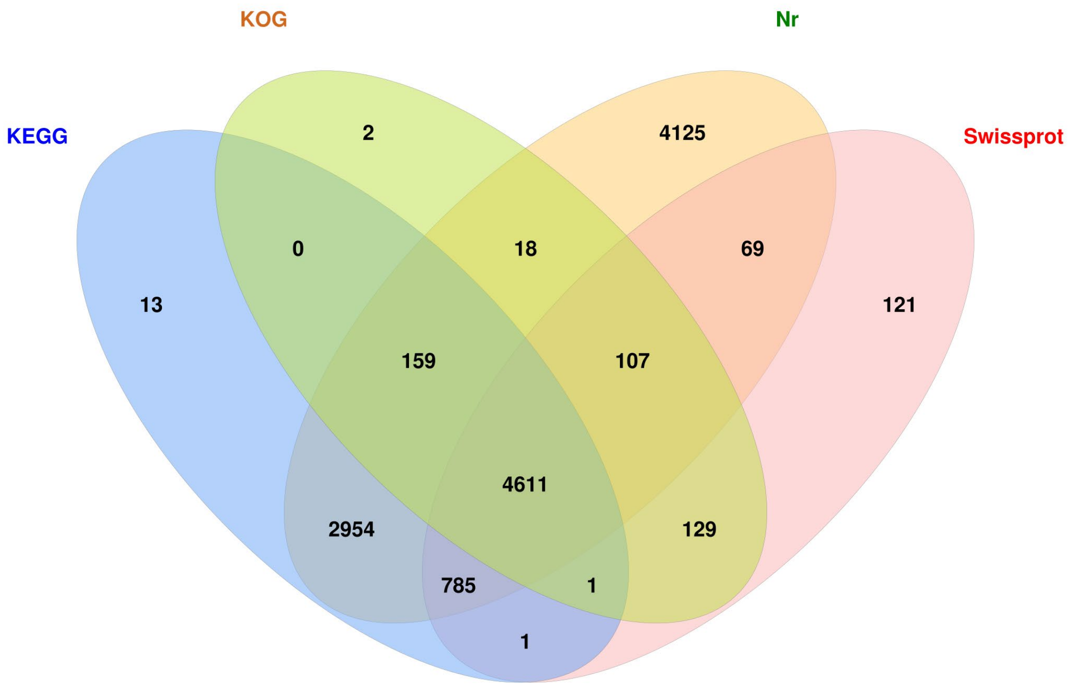

Figure S5. Four major databases annotated with Venn diagrams for the transcriptome of *G. bailinae*.

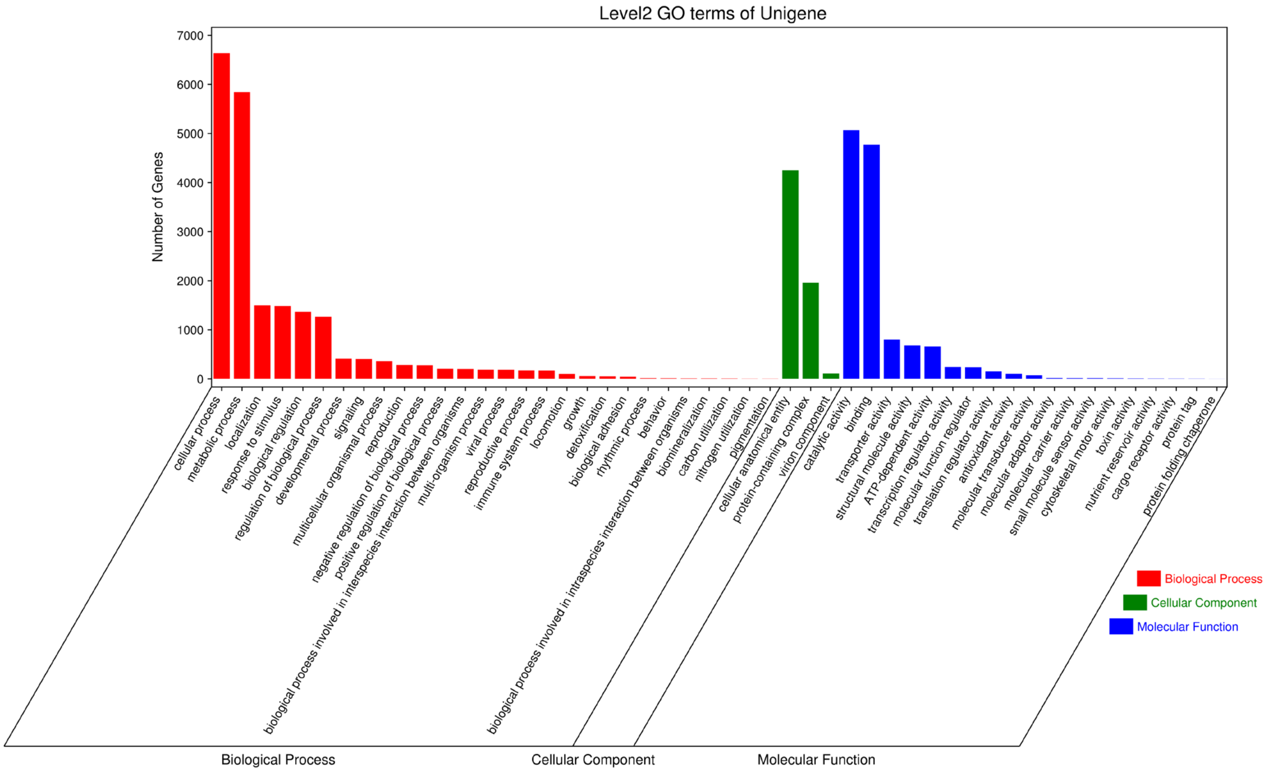

Figure S6. Gene Ontology (GO) Level 2 Classification Statistics for the transcriptome of *G. bailinae*.

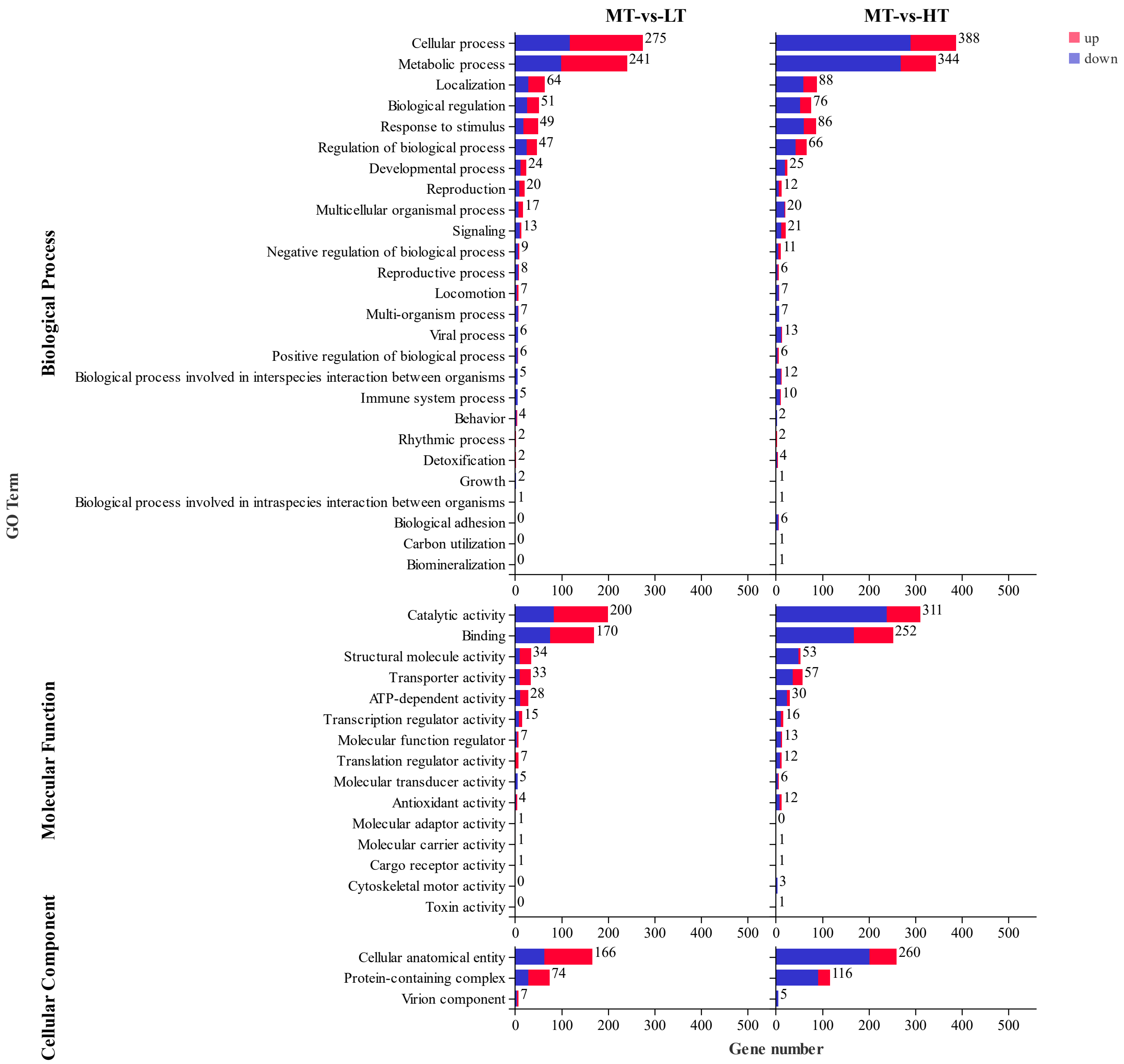

Figure. S7. GO classification of DEGs in MT vs HT and MT vs LT.

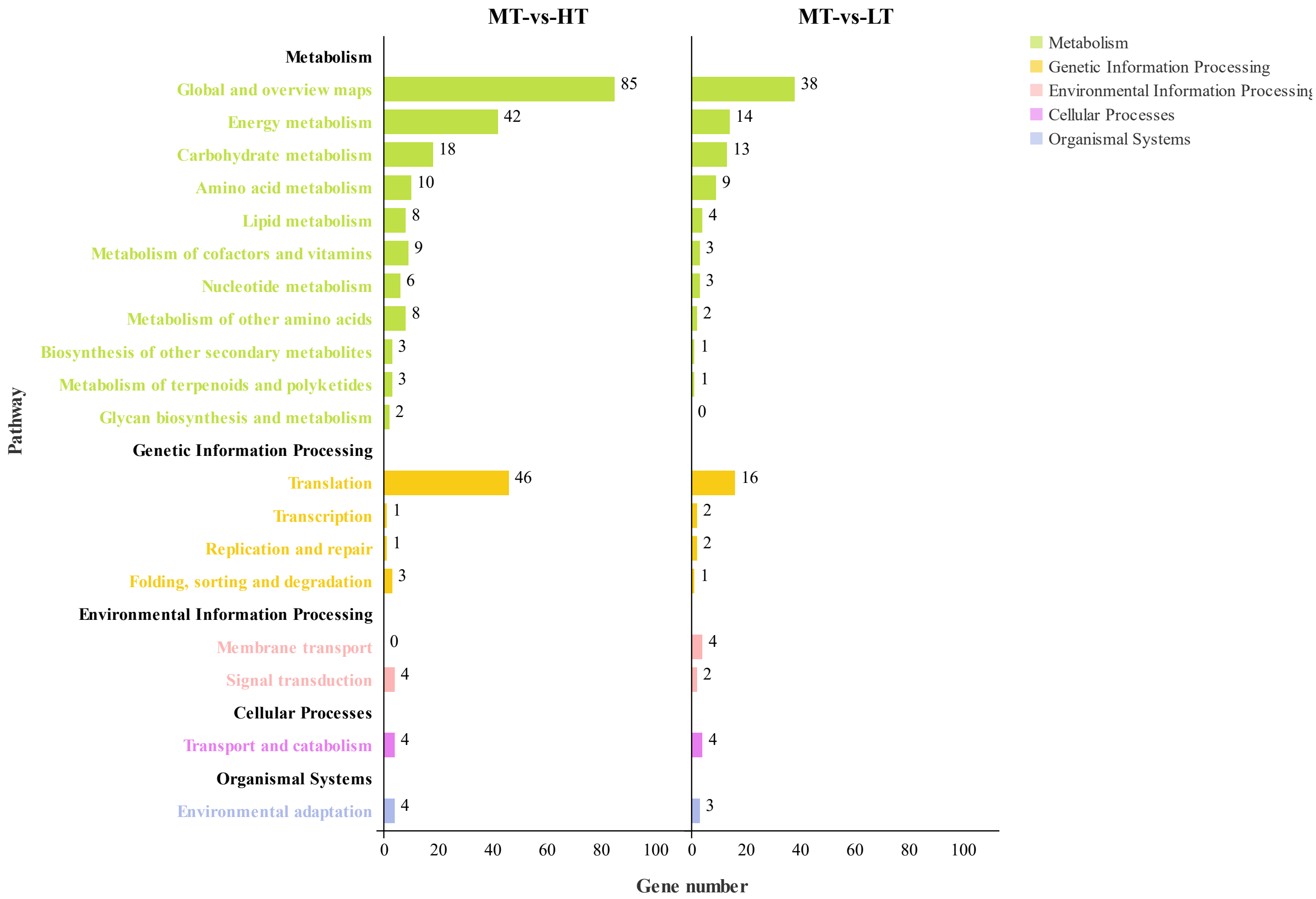

Figure. S8. KEGG classification of DEGs in MT vs HT and MT vs LT.
